# Digestible indispensable amino acid score (DIAAS): 10 years on

**DOI:** 10.3389/fnut.2024.1389719

**Published:** 2024-07-03

**Authors:** Paul J. Moughan, Wen Xin Janice Lim

**Affiliations:** Riddet Institute, Massey University, Palmerston North, New Zealand

**Keywords:** digestible indispensable amino acid score (DIAAS), digestible indispensable amino acid ratio (DIAAR), lysine bioavailability, protein digestibility corrected amino acid score (PDCAAS), protein quality, true ileal amino acid digestibility

## Abstract

The objective of the review is to revisit the findings of the 2011 Food and Agriculture Organization of the United Nations (FAO) Expert Consultation on Dietary Protein Quality Evaluation in Human Nutrition, and to report on progress on uptake of the findings. It is evident that since 2011 there has been a concerted research effort to enhance an understanding of the protein quality of foods. The validity of the growing pig ileal protein digestibility assay has been confirmed and numerous studies reported using the growing pig as a model to give true ileal amino acid digestibility values for foods as consumed by humans. This has allowed for the determination of digestible indispensable amino acid scores (DIAAS) for a range of foods. A new non-invasive true ileal amino acid digestibility assay in humans which can be applied in different physiological states, called the dual-isotope assay, has been developed and applied to determine the DIAAS values of foods. It is concluded that DIAAS is currently the most accurate score for routinely assessing the protein quality rating of single source proteins. In the future, the accuracy of DIAAS can be enhanced by improved information on: the ideal dietary amino acid balance including the ideal dispensable to indispensable amino acid ratio; dietary indispensable amino acid requirements; effects of processing on ileal amino acid digestibility and lysine bioavailability. There is a need to develop rapid, inexpensive *in vitro* digestibility assays. Conceptual issues relating DIAAS to food regulatory claims, and to holistic indices of food nutritional and health status are discussed. The first recommendation of the 2011 Consultation regarding treating each indispensable amino acid as an individual nutrient has received little attention. Consideration should be given to providing food label information on the digestible contents of specific indispensable amino acids.

## 1 Introduction

In 2011 the Food and Agriculture Organization of the United Nations (FAO) convened an Expert Consultation on the subject of “Dietary Protein Quality Evaluation in Human Nutrition.” Fourteen international experts and an FAO Secretariat undertook an in-depth review of aspects pertaining to protein quality evaluation in human nutrition, and the deliberations were published in 2013 (1). The aim of this contribution is to review matters arising from the FAO 2013 recommendations, a decade later. Since 2011, there has been considerable global research effort aimed at improving an understanding of the protein quality of foods.

The 2013 report documented multiple findings, but with two overarching recommendations:

### 1.1 First overarching recommendation

“In dietary protein quality evaluation, dietary amino acids should be treated as individual nutrients and wherever possible data for digestible or bioavailable amino acids should be given in food tables on an individual amino acid basis.”

### 1.2 Second overarching recommendation

“A new protein quality measure known as digestible indispensable amino acid score (DIAAS) is recommended to replace protein digestibility corrected amino acid score (PDCAAS).” “DIAAS can have values below or in some circumstances above 100%. Values above 100% should not be truncated except where calculating DIAAS for protein or amino acid intakes for mixed diets or sole source foods.”

In both cases, it was recommended that the digestibility of each amino acid be given in terms of true ileal amino acid digestibility, and for processed foods where Maillard type damage may have occurred, values for lysine availability (true ileal digestible reactive lysine) should be used. It was recognized at the time of the consultation that there were insufficient published data on the true ileal amino acid digestibility of foods as consumed by humans and rectifying this situation was a key research directive.

In the intervening decade the first overarching recommendation has not received a great deal of attention, possibly because of a primary focus on food scores such as DIAAS. However, it remains an important consideration, especially as further research continues to identify important metabolites associated with specific amino acids, and physiological roles for specific amino acids. The reasoning behind this primary recommendation was firstly that several amino acids have important metabolic fates other than their involvement in protein synthesis and it may be important in this context to have information on the absorbed amount of the amino acid. Secondly, this approach allows for the calculation, where appropriate, of absorbed amounts of conditionally essential amino acids and the dispensable amino acid component. Finally, such data allow for the estimation of the amounts of absorbed amino acids and their adequacy for meeting daily amino acid requirements in the context of meals and dietary patterns. In the latter respect DIAAS values for individual foods are not additive, though true ileal amino acid digestibility values are additive in dietary formulation. Accordingly, it is possible to calculate the DIAAS of a meal or dietary pattern but it is not necessary to do so. DIAAS was designed to meet the need for defining the protein quality of a single food. It gives information as to the ability of that protein to supply available amino acids as if the protein food was the sole source of dietary protein. It is used to compare individual protein sources, particularly for trade purposes and gives a crude estimation of the value of a protein for inclusion in a mixed dietary pattern. Since DIAAS is calculated in isolation from information about the meal or dietary pattern in which it may be consumed, it is necessary to express both amino acid requirements (the reference pattern) and amino acids in the food, relative to protein (the estimated average requirement, EAR, for protein in the case of the reference pattern and the crude protein content of the food in relation to the food amino acids). Although inherently necessary in the case of calculating DIAAS, there are disadvantages in doing this (2).

In the case of ascertaining the adequacy of dietary amino acid intakes in the context of meals or dietary patterns, however, it is not necessary to relate the amino acid contents to protein content. The digestible amino acid contents of the respective dietary proteins in a meal or dietary pattern can simply be multiplied by the amounts of the respective proteins consumed daily (either known or estimated by numbers and sizes of food servings) and each estimated absorbed amino acid intake compared to the required amount. The facility of this approach has recently been demonstrated in the work of Forester et al. (3), who have described a new measure referred to as the Essential Amino Acid-9 Score (EAA-9). Relevant authorities are encouraged to provide newly available information on the digestible amounts of indispensable amino acids on food labels.

The second overarching recommendation has received considerable attention over the past decade with many studies reporting true ileal amino acid digestibility values for a range of foods in a form as consumed by humans, along with the attendant DIAAS values. New methods for determining true ileal amino acid digestibility non-invasively in humans in different physiological states have been developed and animal based ileal digestibility assays have been thoroughly validated. Methodological aspects of DIAAS have been investigated and in some cases aspects of the appropriateness of the DIAAS measure have been challenged. This has occurred largely within a conceptual domain, querying the value of focusing on protein quality to the exclusion of other attributes of a food. These important conceptual issues are discussed. The present overview will mainly focus on this considerable body of work related to DIAAS.

## 2 Protein quality measures

One objective in evaluating dietary protein quality is to predict the contribution of a food protein, or mixture of food proteins, in meeting nitrogen and amino acid requirements for growth and maintenance for people of different ages and physiological states. The extent to which the amino acids from a food or mixture of foods can be used for protein synthesis, when the total intake of utilizable protein is below the upper limit for protein synthesis and when energy and the amounts of other dietary nutrients and co-factors do not limit protein synthesis, is loosely referred to as “protein quality.” Measures of protein quality predict the amount of amino acids from a food that can potentially be utilized for a defined individual and defined physiological state.

Many methods have been developed over the years to enable determination of protein quality (4). Most of these measures are based on biological assays, such as protein efficiency ratio (PER), biological value (BV), net protein utilization (NPU) and net postprandial protein utilization (NPPU), and all these assays have their place.

A more general approach however, has been to estimate protein quality using the chemical score method. Here, a simple model is used to predict the pattern of absorbed dietary amino acids available for protein synthesis, and the estimated amount of utilizable amino acids with reference to an individual’s ideal amino acid balance (usually restricted to the indispensable amino acids) required for body protein synthesis. The chemical score approach has great utility and both the previously recommended scoring method, PDCAAS, and the more recently promulgated DIAAS, are forms of chemical score.

The advantages of DIAAS over PDCAAS have been reviewed in detail (1, 5–8). One important attribute of DIAAS is that it is based on true ileal amino acid digestibility and true ileal reactive lysine digestibility rather than fecal crude protein digestibility, and the true ileal amino acid digestibility assay has been shown in *in vivo* animal studies to accurately predict amino acid absorption and tissue amino acid deposition (9). The limitations inherent in using fecal crude protein digestibility values have been shown in numerous studies including more recent work by Rutherfurd et al. (10) and Mathai et al. (11).

Based on the underlying factors (e.g., type of digestibility measure, amino acid as opposed to crude protein digestibility, lysine availability, non-truncation of score), DIAAS is expected to accurately predict the amount of absorbed first-limiting amino acid supplied by most foods in relation to the requirement for that amino acid, and by implication the amount of utilizable amino acids, whenever the protein requirement is met for a defined person.

DIAAS can be described as:

DIAAS (%) = (mg of available first limiting indispensable amino acid in 1 g test protein)/ (mg of the same amino acid in 1 g reference protein) x 100.
PDCAAS is calculated in the same manner as DIAAS, except that a single value for crude protein digestibility is used to correct gross amino acids to digestible amino acids, and lysine availability is not taken into account specifically. DIAAS is based on updated amino acid reference patterns, and PDCAAS values above 100% are truncated to 100%.

Numerous recent studies have generated DIAAS values for foods and the DIAAS measure has been applied to demonstrate the importance of protein quality in meeting protein and amino acid requirements, and in evaluating the environmental footprints of food production expressed on a protein basis.

## 3 Why is the determination of dietary protein quality important?

### 3.1 Meeting the daily dietary protein requirement in low-income countries and regions

It is frequently assumed that the dietary protein intakes of adults, estimated from population-based food intakes, exceed the safe level of intake (recommended dietary allowance, RDA) for protein [0.83 g protein/kg/day, (12)], even in low-income countries, and that protein is sufficiently supplied. When such observations are made, however, protein is usually given in units of “total” or “gross” protein, with the potential effects of protein quality being ignored. Rather, the safe level of intake for protein, is given in units of available (high-quality) protein, and for a valid comparison, dietary protein intakes should be corrected for the effect of protein quality (13).

The importance of accounting for protein quality is illustrated here by the re-analysis of a published dataset (Source of data: World Resources 2016 Report: see https://www.wri.org/research/shifting-diets-sustainable-food-future) relating “gross” protein intake (population-based) for an adult to the daily protein requirement.

**Figure 1:**
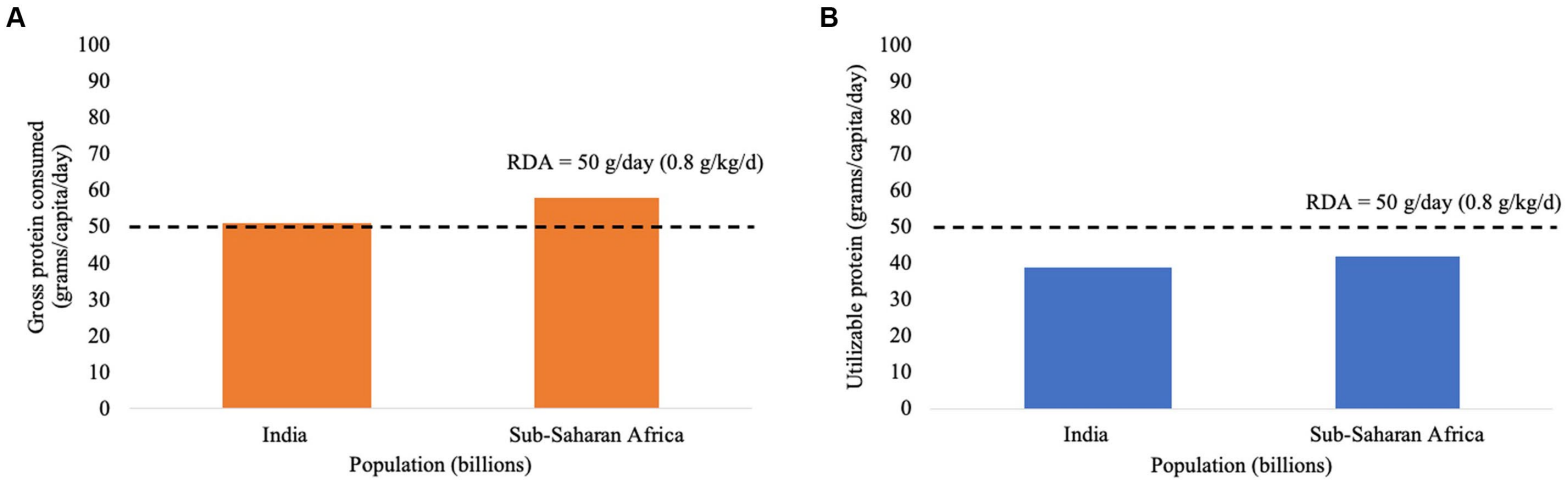
**(A)** Daily protein intake of an adult in India and Sub-Saharan Africa (prior to correction for protein utilizability) compared to Recommended Dietary Allowance (RDA) for protein, as given by World Resources Institute. **(B)** Daily protein intake of an adult corrected for the true ileal digestibility of protein and DIAAS for the diets consumed in India and Sub-Saharan Africa compared to Recommended Dietary Allowance (RDA) for protein, as given by World Resources Institute (DIAAS value for diet based on reference amino acid pattern for the 3-year-old to 10-year-old child).

Daily food protein intakes (based on national food consumption patterns) for India and Sub-Saharan Africa sourced from the Global Agri-WRR model are given in Figure 1A. It is often concluded that in both India and Sub-Saharan African adults receive adequate protein.

These gross dietary protein intakes were then corrected for estimates of dietary protein digestibility (82% for India, and 81% for Sub-Saharan Africa based on data for seven countries) and for DIAAS based on the reference amino acid pattern for the 3-year-old to 10-year-old child as recommended by FAO (1) for application to adults. Lysine was the first-limiting amino and calculated dietary DIAAS values were 93% for India, and 88% for Sub Saharan Africa based on data from seven countries. The corrected protein intakes are shown in Figure 1B.

When the quality of the dietary protein supply is accounted for, the conclusions differ, with protein deficiency now being predicted. This highlights the critical importance of considering protein quality whenever protein intakes are close to required levels. The utility of DIAAS for application in malnourished children in general has been demonstrated by a number of studies including Rutherfurd et al. (14), Manary et al. (15), Manary and Callaghan (16), Shivakumar et al. (17, 18), and De Vries-Ten Have et al. (19), though in one study differences in dietary DIAAS did not relate to growth (20).

### 3.2 Meeting the dietary protein requirement in mid- to high-income countries and regions

In mid- to high-income countries the average adult has a gross protein intake in excess of the RDA for protein and it would appear that protein requirements would be met regardless of protein quality. There is a proportion of the population, however, having low protein intakes and here protein quality can be an important consideration. Moreover, some people (e.g., weight loss, old age, endurance sports) may have higher dietary protein targets than the RDA, which are often accompanied by lower energy intakes. In these cases, protein quality can be important to ensure that the calories derived from protein as opposed to fats and carbohydrates do not become excessive. Both scenarios have recently been evaluated by Moughan et al. (21), with the results highlighting that protein quality can frequently be an important consideration in the diet of consumers in more affluent nations.

As an example of people with lower habitual protein intakes, Sobiecki et al. (22) concluded that UK vegans had an adequate protein intake of 0.99 g/kg/day. If, however, the plant-based diet had an overall utilization value of 70% (typical value for a plant-based diet), the diet would have been protein deficient (0.69 g/kg/day).

In the population at any one time there will be numerous people receiving protein intakes at or below the RDA (23), and protein quality needs to be considered.

What about individuals purposefully targeting protein intakes higher than the RDA and often with accompanying low-calorie intakes? It can be shown Moughan et al. (21) that at a daily energy intake of 108 kJ/kg/day or lower for an average bodyweight US woman, absolute protein intakes of 1.2 g/kg/day or higher combined with low protein quality scores, can lead to protein intake expressed on an energy basis exceeding the recommended upper limit (30%).

The higher the protein intake target and the lower the energy intake, the more pronounced is the effect of DIAAS. In general, for men and women, lower dietary protein quality (DIAAS <100%) can lead to the need for unacceptably high amounts of dietary gross protein required to meet a target for utilizable protein, at energy intakes below around 120 kJ/kg/day and utilizable protein intakes above around 1.2 g/kg/day. Ciuris et al. (24) have also applied dietary DIAAS values to demonstrate the importance of protein quality for vegetarian athletes to reach dietary protein targets.

## 4 Correcting environmental footprint data for effects of protein quality

Life-cycle-analysis (LCA) may be used to quantify environmental outputs associated with different types of food protein production. The environmental measures are often expressed per unit gross protein production with no account being taken of differences in the protein quality of food types. Recently several studies have combined measures of protein quality with LCA results (13, 25–31), and demonstrate the importance of considering protein quality in addition to amounts of protein when evaluating environmental footprint data (13).

The results shown in Table 1 are adapted from the study of Moughan (13) whereby published environmental footprint data (annual freshwater consumption and greenhouse gas emissions; Global Agri Model) are expressed per unit gross protein or per unit digestible lysine to factor in the effect of protein quality. The individual DIAAS values of the food proteins were not applied, as this penalizes some foods as it does not account for the complementarity of food protein mixtures. Food proteins are rarely consumed on their own. Lysine is commonly the first limiting amino acid in mixed diets for humans, and in this case true ileal digestible lysine is a useful surrogate measure for DIAAS. When corrected for differences in protein quality (ability to supply digestible lysine), the rankings of the foods change. When no account of protein quality was made, eggs and pork led to much greater freshwater usage, but when protein quality differences are considered the eggs and pork production actually had the lowest levels of water use. Similarly for the greenhouse gas emissions, eggs and pork had much higher emissions compared to corn on a protein basis, but corn production was a higher emitter than both eggs and pork on a lysine basis.

**Table 1:** Environmental impact of selected plant and animal sources of foods as calculated on a gross protein or digestible lysine basis.

Freshwater		
Food type	1,000 m^3^ per tonne protein	1,000 m^3^ per kg digestible lysine
Wheat	18.43	0.80
Corn	14.22	0.65
Egg	25.80	0.24
Pork	51.60	0.25

Greenhouse gas		
Food type	Tonnes Co_2e_ per tonne protein	Tonnes Co_2e_ per kg digestible lysine
Wheat	78.95	3.43
Corn	105.26	4.79
Egg	263.16	3.99
Pork	339.69	4.09

Adapted from Moughan (13). Original data for impact expressed per metric tonne of protein from Ranganathan et al. (32) and are based on the GlobAgri Model.

The protein quality rating of a food in addition to the gross protein content of the food, should be considered whenever evaluating environmental footprints based on life cycle analyses.

## 5 Development of methods to determine amino acid digestibility and the generation of DIAAS values

### 5.1 Development of isotope-based methods for determining amino acid digestibility in humans

Traditionally “true” and “real” ileal amino acid digestibility have been determined in adult humans based on the collection of samples of ileal digesta from the terminal ileum and with correction for ileal endogenous amino acids. Digesta are collected either through the cooperation of ileostomates or following naso-ileal intubation. These approaches are not straightforward however, nor do they readily allow for investigation of the influence of different physiological states on protein and amino acid digestibility. The latter impediment has been addressed by recent work to develop isotope-based methods for determining amino acid digestibility. Two such approaches have been developed, namely the dual-isotope method and the indicator amino acid oxidation (IAAO) based method.

Both approaches have been the subject of recent review (33–39). To the author’s knowledge neither method for determining amino acid digestibility has yet been fully independently validated over a wide range of foods, but both approaches show considerable promise to allow the generation of digestibility data obtained in humans and to allow investigation of factors such as age, disease state, pregnancy and lactation on amino acid digestibility. The methods have already been applied in multiple studies to give rise to valuable data on ileal amino acid digestibility and dietary DIAAS values. The recent review by Kashyap et al. (38) gives human true ileal amino acid digestibility data obtained using the dual-isotope method for some 20 foods, including a number of foods commonly consumed in low-income countries.

The different approaches to determining ileal amino acid digestibility and availability lead to coefficients of digestibility that have somewhat different physiological meanings (40), but it has been argued that for practical nutrition purposes the different coefficients (true, real, standardized) can be used interchangeably (35).

### 5.2 Development of methods for determining amino acid digestibility in humans using animal models

All of the above-described digestibility assays involving humans are costly, time-consuming and have a high ethical cost, and on their own do not provide a routine method for establishing comprehensive databases of amino acid digestibility in diverse foods. To enable the generation of ileal amino acid digestibility data for foods more generally, animal models for protein digestion in humans have been investigated. Over the last decade considerable work has been undertaken to establish the growing pig as a suitable animal model for protein digestion in humans.

The pig, unlike the rat, has the advantage of being a meal-eating omnivore readily consuming typical foods for humans (41). Protein digestion between the mouth and terminal ileum in the growing pig is similar to that in the adult human from both anatomical and physiological perspectives (42), as is protein digestion between the neonatal pig and human infants (43). It is perhaps not surprising then, that close agreement has been found for ileal protein and amino acid digestibility between pigs and humans (44, 45).

Before concluding that the pig is a valid nutritional model, however, the 2011 FAO Expert Consultation (1) called for further pig/human digestibility comparisons to be made over a wider range of foods. This gave rise to the PROTEOS project funded by sectors of the global food industry and coordinated on their behalf by the Global Dairy Platform, which aimed to further evaluate the growing pig as a model for protein digestion in the adult human, and to use the pig assay to generate true ileal amino acid digestibility data for one hundred foods in the form as consumed by humans. The work has established the growing pig as a replicable and valid animal model Hodgkinson et al. (46), thus providing the means experimentally to establish comprehensive databases on the true ileal amino acid digestibility of human foods.

An interesting development with potential application to both the porcine digestibility assay and to humans has been reported (D Wrigglesworth, U.S. Patent for sampling device, patent 10,993,668, May 4, 2021, patent publication number: 20160038086, assignee: Mars Incorporated). A novel orally-administered device containing protease inhibitors was used to collect samples of digesta (around 400 mg) from the intestinal lumen of normal dogs, and ileal and fecal protein digestibility was compared in poorly and moderately digested protein sources. The digesta collections were successful for 59% of the administrations and showed statistically significant differences for ileal amino acid digestibility between the proteins. Incidentally no differences in fecal digestibility were observed. With further improvements the devices offer a more routine means for obtaining ileal amino acid digestibility data *in vivo*.

### 5.3 Development of *in vitro* methods for determining amino acid digestibility in humans

*In vivo* animal based digestion assays themselves are inherently time consuming and costly, and have a high ethical cost. It is imperative, therefore, that rapid and relatively inexpensive *in vitro* digestibility assays be developed and validated to allow the prediction of true ileal amino acid digestibility in foods. The *in vitro* digestibility assays may be based on either static or dynamic multi-compartment chemico-physical models (47). Much work in this area is currently underway, with results proving promising (48–50).

The *in vitro* assays developed to date are likely to require more refinement to allow general application (47, 51), and should be comprehensively and independently validated. It is also important that they be validated against appropriate *in vivo* data (52).

### 5.4 Generation of DIAAS values using the pig model

Over the last 10 years, and coinciding with its validation, and the publication of a standardized methodology (53), the pig digestion model has been applied widely to generate true ileal amino acid digestibility data and food DIAAS values. A Scopus/PubMed literature search, covering the years 2013 to August 2023, reports more than 250 published scientific papers for the keywords of DIAAS/PDCAAS.

The PROTEOS project has led to a digestibility/DIAAS dataset for 100 foods, and these observations have been augmented by numerous other data generated especially at the University of Illinois [see for example (11, 54–56)], and data for typically eastern foods from the Academy of National Food and Strategic Reserves Administration, China (57–59), and data for common Indian foods (14), and foods in Bangladesh (60), along with data from several other studies including the assessment of novel foods (61–63).

Although the PROTEOS study included some foods typically consumed in African countries, more work needs to be undertaken to determine the true ileal amino acid digestibility and DIAAS values of foods from Africa. There is also the ongoing need to evaluate novel foods that are arising due to the valorization of previously poorly exploited food sources and the application of new technologies.

## 6 Methodological aspects of DIAAS

Several studies have been published addressing methodological aspects related to the DIAAS measure (2, 3, 64–67).

### 6.1 Reference essential amino acids and their normalization

The amounts and patterns of each indispensable amino acid (IAA) in the reference protein used to calculate DIAAS reflect the amounts considered to meet the daily requirement for each amino acid following the consumption of an amount of the protein equal to the EAR. The reference protein can be viewed as providing an “ideal amino acid balance” such that each IAA is provided in the correct amount and balance in relation to the other IAAs and to the dispensable (non-essential) amino acid component (sum of the dispensable amino acids, DAAs). In practice, however, the reference amino acid pattern is given as estimates of daily IAA requirements expressed on a protein basis. It is normalized by the EAR for protein. It is important to realize, therefore, that the pattern used is not an empirically derived ideal amino acid balance taking into account optimal ratios between individual IAAs and the indispensable and dispensable components, but rather is a composite of daily amino acid requirements and an estimate of the daily protein requirement that have been determined using different approaches.

It is uncertain, therefore, as to whether the reference pattern ratio of IAAs to DAAs is accurate in the context of an ideal amino acid balance. In fact, when compared to ideal IAA/DAA ratios found in other simple stomached mammals the current ratio would appear to be low (2).

If an IAA is first limiting, the DIAAS value reflects this, if however, all of the IAAs are found in a protein in excess of the required amount (no individual IAA is limiting) it is assumed that the excess IAAs are mainly transaminated to DAAs post-absorption and along with the synthesis of dispensable amino acids from ammonium absorbed from the gut, the DAA component is not limiting. Based on this assumption and in recommending DIAAS, it was held that in practice the DAA component is not limiting and that the DIAAS calculation is restricted therefore to the IAAs. It is pertinent to note that although true ileal digestibility is the best approach for predicting the uptake of amino acids during digestion, estimates of fecal crude protein digestibility are needed to model overall nitrogen transactions in the body.

Recently, Adhikari et al. (68) have addressed the potential importance of the DAA fraction, and have modelled the potential effects of the DAA component of a protein, and assumptions around the extent of transamination, on DIAAS and predicted utilizable protein.

While it appears likely that specific DAAs may become limiting in humans under certain conditions (69), it is unclear as to the potential effects of less than “ideal” amounts of the dietary DAA component in total. It is usually assumed that in practice the DAAs are not limiting for protein synthesis. It has been shown, for example, in clinical studies with humans that the ingestion of IAAs alone stimulates muscle protein synthesis equivalently to a mixture of the same amount of IAAs supplied along with additional DAAs (70). DAAs are required for protein synthesis, so when the IAAs were given alone the DAAs were presumably obtained from endogenous sources and from the recycling in the gut of ammonium from blood urea. Ingestion of a mixture of DAAs on their own failed to stimulate muscle protein synthesis. In contrast to these findings nitrogen balance studies have shown an effect of the DAA component on the efficiency of utilization of the IAAs (71).

Regardless, the DAA component does remain an important consideration in the context of DIAAS. A higher IAA/DAA ratio in an ideal amino acid pattern has a large absolute effect on the estimated DIAAS value (2). More accurate estimates of the optimal IAA/DAA ratio and better harmonization between the IAA requirements and the EAR for protein has the potential to increase the accuracy of DIAAS values. An accurate estimation of DIAAS relies on accurate and compatible estimates for both the individual IAAs and the EAR for protein.

### 6.2 Accuracy of IAA requirement values

The accuracy of the current estimates of IAA requirements used for determining DIAAS has been queried (1, 36, 72, 73). The current estimates are based on a limited number of studies, and often may provide minimal values rather than requirements to optimize organ and body function (74). Further, their generality in application to people in different physiological and nutritional states is also in question. Estimated amino acid requirements are usually given as population averages for a person of defined age (e.g., infant, child, adult) or physiological state (e.g., pregnant, lactating mother). In reality, however, amino acid requirements are influenced by multiple factors (e.g., age of adult, disease and nutritional status, surgery, diet composition) and are dynamic rather than static values (15, 75–77). There is a paucity of amino acid requirement estimates for people in these different physiological and nutritional states.

More and better information on individual amino acid requirements and optimized IAA profiles would lead to enhanced accuracy and versatility in the DIAAS measure. Inaccuracy in the estimated IAA requirements has the potential to affect absolute DIAAS values, but also relative DIAAS values calculated across foods, because the first limiting amino acid differs among foods and any inaccuracy in requirement estimation may vary among the IAAs (2).

### 6.3 Conversion of nitrogen to protein in foods

In calculating the DIAAS values for a food, each digestible IAA in the food is expressed per unit crude protein which is determined by multiplying the nitrogen content of the food by the generalized conversion factor of 6.25 (78). This approach has been criticized, as using the generalized conversion factor rather than specific factors for each food, can lead to both overestimation and underestimation of DIAAS. This is true, but food nutrient systems need to be consistent, and if a food specific conversion factor is applied in calculating DIAAS the same factor should be applied to determine the gross food protein intake. The end result is that the estimate of utilizable protein for the food does not differ greatly. Craddock et al. (66) raised this issue in relation to almonds that have a specific conversion factor of 5.2, considerably lower than the generalized factor. If it is assumed that almonds have a digestible lysine content of 5.87 mg/g dry matter (first limiting amino acid) and a nitrogen content of 44 mg/g dry matter, it can be shown that the calculated DIAAS value (6-month to 3-year-old child reference pattern) is 38% using the generalized factor and a considerably higher 45% using the food specific factor. If, however, the factors are also applied consistently to nitrogen intakes from a single serving of almonds (30 g), the differences in estimated utilizable protein intake per serving are negligible (2.26 g when the DIAAS of 38% and the consistent factor of 6.25 were used, and 2.23 g when the DIAAS of 45% combined with the consistent factor of 5.2 were used). Only when the conversion factor is used inconsistently (the low conversion factor of 5.2 is used to generate DIAAS but the higher factor of 6.25 is used to calculate the utilizable protein intake per serving), a higher estimate (2.68 g/serving) is found for utilizable protein intake per serving.

An even more accurate estimate of utilizable food protein intake would be found by correcting food nitrogen content for its non-protein nitrogen content before converting the proteinaceous nitrogen to crude protein using the food specific nitrogen to protein conversion factor, and then calculating DIAAS using the food specific factor to determine the crude protein content of the food. If specific food nitrogen to protein conversion factors are to be used, however, they need to be applied consistently, and this would require the protein contents of foods to also be based on the specific food factors. Conversely to the direction in the DIAAS values, the stated protein content of some plant proteins would decrease and the protein content of some animal-sourced proteins would increase.

The added complexity in the calculations needs to be weighed against any improvement made in the accuracy of the final estimate of utilizable protein intake.

### 6.4 Processed foods

Processing (e.g., soaking, heating, extracting, extruding) of raw foods often leads to increases in protein and amino acid digestibility as discussed by Craddock et al. (66), especially in plant-based foods where the treatment may deactivate antinutritional factors (ANFs) and lead to beneficial structural alterations in the complex food matrix. It is for this reason that in the PROTEOS study and other recent work to determine true ileal amino acid digestibility in foods, it was ensured that the foods studied were in the form as consumed by human subjects rather than in the raw form. This is an important consideration. An advantage of DIAAS in this respect is that the true ileal amino acid digestibility assay has been shown to be more sensitive than fecal measures of digestibility for detecting changes in amino acid digestibility due to the effects of food processing and ANFs (79, 80).

It is not always the case, however, that the processing of foods enhances protein digestibility and there is an extensive literature documenting deleterious effects of food processing (especially heating and drying) on the amounts of an amino acid (67) and amino acid digestibility and availability, due to complex Maillard-type reactions that can occur under some processing conditions, and during food storage (81, 82). The nutritionally important amino acid, lysine, is particularly susceptible to structural alterations leading to lowered bioavailability (77, 83, 84). As a consequence of this, a lysine bioavailability assay (based on the digestibility of reactive lysine) has been developed (85) and is integral to the calculation of DIAAS for foods susceptible to damage during processing. This step in calculating DIAAS values has been largely overlooked, but is important in describing protein quality (DIAAS) in foods where the protein has been damaged by processing. For some foods, differences between lysine digestibility and availability and thus the calculated DIAAS, can be quantitatively significant (Table 2). DIAAS takes into account the effects of processing, at least to some extent.

**Table 2:** Mean true ileal digestible total (conventional analysis) and reactive lysine contents (g/kg air-dry) in selected foods.

Food	Digestible lysine^a^		% Difference^d^
	Total^b^	Reactive^c^	
Collagen	36.0	36.0	0
Cooked black beans	13.2	11.3	14.4
Cooked pigeon peas	17.0	16.7	1.8
Heated peas	9.5	8.8	7.4
Split peas	16.1	15.4	4.3
Processed wheat bran	1.9	1.5	20.9
Toasted wheat bread	2.1	1.4	33.7
Wholegrain bread	2.4	2.0	16.7
Popped rice cereal	0.7	0.3	57.1
Grain-based cereal	1.2	0.5	58.3
Whey protein isolate	82.8	82.7	0.1
Heated skim milk powder	19.8	16.6	16.2
Skim milk powder	19.8	16.6	16.2
Whole milk powder	26.2	24.0	8.4
Lactose-hydrolyzed milk powder	27.2	25.1	7.7

Adapted from Hodgkinson et al. (86) and Moughan et al. (87).

a Determined in the growing pig or growing rat; from Rutherfurd and Moughan (116).

b Based on conventional amino acid analysis.

c Based on determination of reactive o-methylisourea lysine in diet and ileal digesta.

d % difference = (Total – Reactive)/Total x 100.

Amino acids other than lysine (arginine, methionine and cysteine, threonine and tryptophan) are also subject to structural changes that can affect their bioavailability (88). These amino acids deserve more attention in this context, and bioassays similar to the digestible reactive lysine assay should be developed. However, lysine is the most susceptible amino acid to damage and loss of availability during food processing, and is a sensitive monitor for generalized protein damage. It is also often the first-limiting amino acid in diets.

### 6.5 Inadequate quantum of ileal amino acid digestibility data

When DIAAS was first introduced FAO (1), a dataset of true ileal amino acid digestibility for some 180 foods was collated (89), see https://www.fao.org/ag/humannutrition/36216-04a2f02ec02eafd4f457dd2c9851b4c45.pdf, but was considered at the time by the Expert Consultation to not be comprehensive enough to allow for the practical implementation of DIAAS. In the interim, other bodies and groups (90, 91) and commentators (3, 30, 64, 92–94) while recognizing the strengths of DIAAS, have also called for the generation of more data on ileal amino acid digestibility.

In the deliberations of the FAO Consultation the global food industry was urged to support research into the ileal amino acid digestibility of a wider range of human foods. This gave rise to the PROTEOS project, involving the cooperation of researchers from four universities and was completed in July 2023. This work has led to the generation of true ileal amino acid digestibility coefficients for a further 100 foods and over a wide range of food groups. It is estimated that other published studies conducted over the last 10 years have generated ileal digestibility data for at least a further 230 foods. Collectively there are now true ileal amino acid digestibility data well in excess of 400 foods including a broad range of plant-based foods including fruits and vegetables.

Moreover, the animal nutrition-based literature provides copious data on the effects of processing on ileal amino acid digestibility. Much of this information can be translated within a human food processing context. There appears to be a surprisingly low overall degree of variability for true ileal amino acid contents and DIAAS within a human food but across multiple factors (e.g., cultivar., batch and sometimes processing or cooking method) (95), suggesting that for regulatory purposes the application of overall fixed conservative digestibility estimates and DIAAS values for foods and food types may be acceptable, as suggested by Marinangeli and House (64). Such food values could be adjusted up or down based on determined *in vitro* digestibility estimates.

There is a need to bring these comprehensive ileal amino acid digestibility data together into a single readily accessible database.

### 6.6 Ethical cost of animal and human digestibility assays

True ileal amino acid digestibility can be determined in humans, and importantly the newly developed dual-isotope digestibility assay gives the means of determining ileal amino acid digestibility in humans of different ages and physiological and disease states. This will be valuable for enhancing an understanding of protein digestion in humans. There is, however, a high ethical cost involved in human research and no human-based assay can be considered routine. Some (64) have also highlighted the ethical cost and increasing opposition around using animal-based digestibility assays such as those involving rats and pigs. It is anticipated that in the future the animal-based ileal digestibility assays will be used to provide generalized tabulated digestibility estimates for foods and food groups and may be applied to generate information on novel foods, but that such assays will increasingly give way to more rapid, non-invasive *in vitro* assays for the routine evaluation of foods.

### 6.7 Additivity of DIAAS values in meals and diets and associative effects in whole meals

DIAAS was developed to provide information about the amount of the first limiting amino acid supplied relative to the required amount for that amino acid in a protein source, when that protein is ingested at an amount to meet the EAR for protein. A DIAAS of 100% means that each IAA exactly meets the required amounts of IAAs; a DIAAS less than 100% means that one or more of the IAAs are limiting for protein synthesis and the score gives the degree to which the first limiting amino acid is undersupplied relative to the required amount; a DIAAS greater than 100% means that the IAAs are supplied in excess of 100%. DIAAS provides valuable information. If a protein with a DIAAS <100% is ingested as the sole food, the protein will not be fully utilizable and the score provides information about which amino acid needs to be supplied from other foods to enhance utilizability. A protein with DIAAS >100% will be highly utilizable if ingested alone, but can be combined with other proteins to have a complementary effect on the intake of the IAAs. Information provided by DIAAS is practically useful.

In calculating the DIAAS values, the ratio of the amount of each IAA relative to its requirement is calculated (digestible indispensable amino acid ratio, DIAAR), and in addition to DIAAS the DIAAR values themselves provide useful information (65, 67). Table 3 shows the DIAAR calculated for a whey protein isolate. Histidine is supplied at the lowest level (DIAAR = 1.09), but all of the dietary IAAs are estimated to be supplied in excess of requirement. The whey protein isolate may be used to complement amino acid supplies from other proteins that may be limiting in IAAs. The DIAAR values, however, provide additional information. In the case of whey for example, the protein supplies particularly high amounts of tryptophan and leucine, amino acids that have important physiological roles in addition to being building blocks for protein synthesis. The latter information is lost in the single score.

**Table 3:** Digestible indispensable amino acid ratios (DIAAR) for a whey protein.

Amino acid isolate	DIAAR^a^
Threonine	1.80
Methionine + Cysteine	2.29
Valine	1.21
Isoleucine	2.22
Leucine	2.57
Tyrosine + Phenylalanine	1.71
Histidine	1.09
Tryptophan	3.35
Lysine	2.51

a Digestible indispensable amino acid ratio. Based on reference amino acid pattern for the 3-year-old to 10-year-old child.

It is important to note that DIAAS values are not necessarily additive, and if information about the DIAAS of a meal or dietary pattern is required this should be calculated based on the amount of each true ileal digestible amino acid supplied by each respective food protein. True ileal amino acid digestibility values are additive across different food proteins. In the case of meals and dietary patterns, however, it is not necessary to calculate DIAAS *per se*. DIAAS was designed to have a specific application to single protein sources. For meals and dietary patterns, the amounts of each IAA provided relative to the daily requirement can be calculated from first principles based on amounts of foods ingested, amino acid contents and the true ileal amino acid digestibility for each food. This is one of the reasons why the FAO (1) Expert Consultation recommended, first and foremost, that information should be provided for all foods on the ileal digestible amount of each IAA provided by a food, and that each amino acid be regarded as a nutrient in its own right. This does not diminish the importance or application of DIAAS values, but rather highlights the need for complete information (including data on the digestible amino acid contents) on food proteins.

The possibility of associative effects between foods has been discussed in relation to true ileal amino acid digestibility (66, 96).

Whereas holistic properties of foods involving the entire food matrix are undoubtedly important and food interactions can influence nutrient uptake and utilization (92, 97–99), the importance of the overall effect of associative interactions on amino acid digestibility in the context of normal meals may be somewhat overstated, as true (standardized) ileal amino acid digestibility values have been shown in several studies to be broadly additive over a wide range of foods (100–103). This is expected to be particularly so for most foods consumed by humans and given the form in which they are consumed, where ANFs and plant fiber levels are usually relatively low compared to feedstuffs for animals, whereby the additivity of true ileal amino acid digestibility has been demonstrated.

Where a significant associative effect is suspected, and there may be situations where this arises, amino acid digestibility should be determined for the combination of proteins provided as a meal, and the use of *in vitro* digestibility assays may be particularly useful here to determine relative changes in digestibility.

## 7 Conceptual aspects concerning DIAAS

There has been some discussion, not so much questioning the scientific accuracy of DIAAS, but rather addressing conceptual issues around its application in practice particularly in respect of jurisdictional regulatory frameworks and public health outcomes in wealthier countries.

Marinangeli and House (64) have discussed practical implications of a transition from PDCAAS to DIAAS in industrialized food systems. They note that for several plant-based foods although there are differences between PDCAAS and DIAAS the differences are not always great (2–13% for the limited number of foods in the one study quoted). Given this possibility, the authors appropriately question the practical advantage and cost implications of transitioning to DIAAS.

Although, in general PDCAAS undervalues the protein quality of animal-sourced foods and overvalues plant-sourced foods, it is the case that the differences between PDCAAS and DIAAS are not always high. Nonetheless there are many instances where the difference is of a practically significant magnitude, such as the difference between DIAAS and PDCAAS for unprocessed soya products (DIAAS 86% versus PDCAAS 92%) (81), as well as other plant-based foods (104). This is further illustrated by the data shown in Table 2, and the fecal and ileal digestibility data presented by Adhikari et al. (67).

If the global food supply shifts more towards plant and away from animal protein, as is widely proposed, the need for accurate estimates of protein quality will be even more important, and this may be further exacerbated by enhanced atmospheric carbon dioxide levels potentially leading to lower plant protein contents (105).

The need for accurate estimates of protein quality in the latter context is brought into focus by the recent work of Conzuelo et al. (106) involving modelling the protein quality of daily food patterns as recommended for the “planetary health diet” developed by the EAT-Lancet Commission. For the recommended lower-quality daily dietary patterns, estimated protein quality was low (DIAAS 71 and 76%) and there were large differences between PDCAAS and DIAAS (e.g., DIAAS 76% versus PDCAAS 88%). When higher protein quality foods were added to the pattern, DIAAS was still below 100% (DIAAS 88 and 94%), and practically significant overall differences between PDCAAS and DIAAS (e.g., DIAAS 83% versus PDCAAS 88%) persisted.

Conceptually, a DIAAS-based system of protein quality evaluation mirrors a PDCAAS-based system. The only difference is that DIAAS follows current best practice in describing amino acid requirement patterns and amino acid availability, and thus offers more accurate estimates of protein quality. This is of crucial importance in low-income countries and there is an argument for having one harmonized global system for describing protein quality. In high income countries there may not always be the same imperative around protein quality as is the case in developing nations, but many of these developed economies not only consume the food proteins they produce internally within the economy but also export them widely.

With a new protein quality metric, inevitably the protein quality values and rankings of different foods change to some extent. It is important, therefore, to evaluate if this may lead to unintended consequences in practice. A particular concern relates to food sources of protein that qualify for a protein content claim under PDCAAS but would be ineligible under DIAAS. Is there a risk that the positive attributes of some relatively protein-rich plant-based foods could be downplayed if they have DIAAS scores lower than their PDCAAS, and much lower DIAAS than for animal-sourced foods?

This has been evaluated in the study of Sa et al. (107) for lentils, an important protein source. The authors conclude that with PDCAAS and US standards, lentils qualify for a “good source” claim for protein, but with DIAAS and following the FAO (1) recommendations, such a claim could not be made. The authors discuss how this outcome relates to several plant-sourced foods (e.g., navy beans, yellow peas, tofu) and make the point that with the promulgated DIAAS system, several foods from the categories seeds, nuts and pulses would disappear from the 2019 Canadian Food Guide’s ideal plate, which would be inconsistent with current food guidelines. Similar conclusions were drawn in the study of Cargo-Froom et al. (108).

This, however, is not a criticism of DIAAS itself, which is merely a more accurate means of describing protein quality, but relates more to the FAO proposed regulatory system and the cut-off points for making claims. The two components, metric and system, should not be conflated as part of the same issue. Simply because a certain protein fails to make a claim under a particular proposed system, this should not be used as a criterion to judge the suitability of the protein quality metric. Nevertheless, it remains a concern that there may be unintended consequences in adopting the proposed system for making claims. In this respect, it is important to note that the FAO (1) recommendations on the regulatory aspects of DIAAS were couched as guidelines with the suggested cut-offs only given as examples, and it was stated in the report that: “the DIAAS cut-off points in the context of making claims require careful further consideration (e.g., in relation to national and local dietary patterns)” (1). The Expert Committee recommended the development of a published set of guidelines for Industry. It would appear timely to devote attention to the development of such a set of guidelines that would be acceptable and relevant across multiple jurisdictions, and would take into account specific attributes of foods in the context of providing protein and amino acids.

A bigger picture concern with DIAAS is that because in general DIAAS gives higher scores for animal-based proteins and foods than PDCAAS, leading to designations such as “excellent source,” this may encourage the consumption of animal-based foods which may in turn have negative consequences for both the environment and public health (64, 96, 109). The roles of animal-based foods in both latter respects, however, are contentious. Moreover, DIAAS values are restricted to providing information on the delivery of the most limiting amino acid in a food, meal or dietary pattern, and should not be interpreted to mean anything more or less than this. This is important information in its own right, and is restricted to the domain of protein quality.

Many animal and plant foods will be rich sources of other essential nutrients and beneficial compounds (104, 110–112), and may also have specific beneficial holistic properties, while others may contain ANFs and other compounds considered to impair health and function, but a high or low DIAAS in its own right should not be interpreted as providing any information on such properties. Perhaps the somewhat emotive terms such as “poor,” “good,” “excellent” used in describing protein quality should be replaced by more descriptive and restrictive terms such as “low,” “medium,” “high” and “complementary.” Humans consume foods, not proteins, and the information provided by DIAAS should be restricted to the protein component of a food.

More overarching food and diet quality scores have a place in public health nutrition (109, 113–115), but the components of these scores should not be conflated with protein quality metrics. It remains that consumers and industry require information on protein quality *per se*, and the ability of a particular food to provide utilizable protein, and in this context, it is argued that DIAAS provides that information most accurately. Consumer education and food regulation need to ensure that information on protein quality and other important information on attributes of a food are conveyed to consumers in such a manner as to allow informed decisions. Protein quality metrics should not be used or promoted as proxies for overall food quality attributes, rather the information they convey should relate solely to the estimated delivery of IAAs. It is argued that protein quality is one standalone set of useful information, with a specific purpose.

## 8 Conclusion

Currently DIAAS is the most accurate means to routinely give a single protein quality value for a stand-alone food. This should not be taken to mean that DIAAS is a perfect measure, and in fact considerable scope exists to improve the accuracy of DIAAS values. Careful consideration should also be given as to how DIAAS is applied in relation to food regulations to ensure that use of the metric does not lead to unintended consequences and misleading representations of certain food types.

The amino acid delivery of meals, dietary patterns and personalized meal plans are best assessed by the direct application of food amino acid contents, and true ileal amino acid digestibility and availability coefficients. For this reason and given the growing importance of having information on the delivery of individual amino acids related to specific physiological roles, consideration should be given to providing information in food labelling on digestible amino acid contents.

True ileal amino acid digestibility coefficients have been shown empirically to be accurate estimates of amino acid absorption in most cases. They have also been shown to be sensitive indicators of changes to proteins incurred during processing and storage. Further, and because such coefficients include relevant corrections for endogenous ileal amino acids, they reflect the effects of most common plant ANFs. None of these claims can be made for the outmoded fecal crude protein digestibility measure. If the intention is to use the world’s protein resources more efficiently and to describe available amino acid levels as accurately as possible, then a shift in practice to using DIAAS and ileal amino acid digestibility is a major step forward.

## Author contributions

PM: Conceptualization, Writing – original draft, Writing – review & editing. WL: Writing – original draft, Writing – review & editing.
